# Postvoid Residual Thresholds Used to Define Chronic Urinary Retention: A Systematic Review

**DOI:** 10.1016/j.euros.2026.06.010

**Published:** 2026-07-03

**Authors:** C.H.H. Christiaans, J. de Klerk, A.K. van Velsen, B.F.M. Blok

**Affiliations:** Department of Urology, Erasmus University Medical Center, Rotterdam, The Netherlands

**Keywords:** Benign prostatic hyperplasia, Chronic urinary retention, Postvoid residual, Underactive bladder, Urinary retention

## Abstract

**Context and Objective:**

Chronic urinary retention (CUR) is a clinically relevant but poorly defined urological condition, often assessed in the clinic using postvoid residual (PVR) volumes. The lack of consensus on the PVR threshold that defines CUR limits diagnostic consistency, clinical decision-making, and comparability of research findings. This review aims to systematically examine how CUR is defined in the current literature, focusing on the reported PVR thresholds.

**Evidence acquisition:**

In this systematic-narrative review, a search of five databases (MEDLINE, EMBASE, CENTRAL, Web of Science, and Google Scholar) was conducted in August 2022 and December 2025. Eligible studies included randomized trials, observational studies, and systematic reviews that defined CUR using a PVR threshold in adult patients.

**Evidence synthesis:**

A total of 4588 abstracts were screened across two searches, resulting in 14 inclusions. Eleven studies focused on patients with CUR due to benign prostatic hyperplasia. PVR thresholds for defining CUR varied between 150 ml and 1000 ml, with 300 ml being the most reported in 10 studies. Seven studies reported on patients who were able to void, three of which also reported on patients who were unable to void. The remaining seven studies did not report the voiding status of the participants. Four studies repeated the PVR measurements. Six studies used ultrasound or a bladder scan to measure PVR.

**Conclusions:**

This review highlights the inconsistency in the definitions and PVR thresholds used to define CUR in the literature. The available evidence was limited and heterogeneous, with substantial variation in study populations, voiding status, and measurement methods. Therefore, the current literature does not provide sufficient consistent evidence to support a single definitive PVR threshold for CUR.

## Take Home Message

The definitions used for chronic urinary retention (CUR) in the literature vary substantially, with different postvoid residual thresholds, measurement methods, and patient populations. A standardized, patient-specific definition of CUR that takes into account individual risk factors could improve treatment algorithms and facilitate future urological research.

ADVANCING PRACTICE **What does this study add?**

Chronic urinary retention (CUR) is a common but poorly defined medical condition. In the clinic, it is most often defined by postvoid residual volume (PVR). This study shows large variability in PVR thresholds and definitions used for CUR. A risk-stratified, patient-specific definition could allow for more accurate identification of patients at risk for treatment and facilitate urological research.

**Clinical Relevance**

This review highlights the need to move beyond a fixed PVR threshold when defining chronic urinary retention. Although a threshold of ≥300 ml is commonly used and aligns with contemporary consensus definitions, PVR alone is an imperfect surrogate because it does not account for symptom burden, risk of upper tract deterioration, recurrent infections, bladder capacity, neurogenic status, or measurement variability. A standardized, patient-specific, risk-stratified definition could improve diagnostic consistency, guide treatment decisions, distinguish patients requiring active intervention from those suitable for surveillance, and improve comparability across future urological studies. Associate Editor: Véronique Phé, M.D., Ph.D.

**Patient Summary**

In this review, we looked at how chronic urinary retention is defined in the literature. We found that definitions varied widely in the literature, and no one definition could be concluded. A definition based on risk factors could improve treatment for patients with chronic urinary retention.

## 1 Introduction

Urinary retention (UR) is a common but complex urological condition, broadly defined as the inability to partially or completely empty the bladder. This can be divided into acute or chronic UR, depending on the duration [1]. Acute UR (AUR) is defined as a painful, palpable, or percussible bladder in a patient who is unable to pass urine. Chronic UR (CUR) is defined as a nonpainful bladder that remains palpable or percussible after the patient has passed urine [1], [2].

CUR may cause lower urinary tract symptoms (LUTS), urinary tract infections (UTI), bladder stones, urinary incontinence, and elevated intravesical pressures, which may lead to hydronephrosis and renal failure [3]. Timely diagnosis of CUR is crucial to prevent such complications.

Postvoid residual (PVR) volume is often used in clinical practice to define CUR. However, the volume that would be considered CUR remains unclear and is not incorporated into most definitions [4]. In addition, PVR is dependent on the time after voiding and the maximal bladder capacity [5]. This makes it difficult to determine the true prevalence and associated risks, limits the ability to perform epidemiological studies, and creates uncertainty in clinical decision-making, particularly when it comes to choosing appropriate intervention thresholds.

To address these challenges, a better understanding of how CUR is defined in the literature is needed. This review aims to systematically identify and synthesize the definitions of CUR used in the medical literature, focusing on reported PVR thresholds in both male and female. By clarifying how CUR is defined, this review may contribute to greater consistency in diagnosis and reporting, inform future research methodology, and support the development of standardized clinical guidelines for the management of CUR.

## 2 Methods

### 2.1 Review design

A systematic-narrative review was conducted [6]. In this format, the search strategy and inclusion and exclusion criteria were guided by systematic review principles. The synthesis and analysis of the selected studies were done narratively. The protocol of this study was registered in the online systematic review register PROSPERO (CRD420251076301). The study was performed according to the Preferred Reporting Items for Systematic Review and Meta-Analyses statement (PRISMA) guidelines [7].

### 2.2 Search strategy

An information scientist designed and conducted an electronic search in Embase, Medline ALL via Ovid, Web of Science, Cochrane Central Register of Controlled Trials, and an additional search using Google Scholar on August 12, 2022, with an updated search on December 11, 2025. The search strategy included terminology related to UR and PVR (Supplementary material). The searches were restricted to articles published in English.

### 2.3 Eligibility criteria

Eligible study designs included randomized controlled trials, nonrandomized intervention studies, systematic reviews, and observational studies. Studies were eligible if they focused on CUR in adults and explicitly defined CUR using a PVR threshold. To ensure quality and relevance, only peer-reviewed, full-text articles in English with a sample size >10 participants were included.

### 2.4 Study selection

Study selection was conducted in two phases corresponding to the two search periods.

Two reviewers (J.K. and R.C.) independently screened abstracts of all citations and full texts in the first screening period. Three reviewers (C.C., J.K., and A.V.) independently screened abstracts of all citations and full texts in the second search period. The objective of this study was to identify PVR-based CUR definitions reported in the literature. As this objective is descriptive in nature and not aimed at synthesizing treatment effects or causal associations, the methodological quality of individual studies was not expected to influence the findings. Therefore, a critical appraisal was deemed unnecessary and was omitted from the review process.

### 2.5 Data extraction and synthesis

Data extraction was performed independently by three reviewers (C.C., J.K., and A.V.). Discrepancies were resolved by discussion with an experienced investigator (B.B.) Included studies were analyzed in both narrative and tabular formats, encompassing their study design, year and country of publication, number of participants, inclusion period, condition, population age, baseline PVR, definition of CUR in PVR, and method of PVR measurement. For the included systematic review, only the reported CUR definition was extracted, as patient- and study-level characteristics were not attributable to individual patients or primary studies, and to prevent potential overlap with primary studies included separately in this review. A meta-analysis was not performed, as the objectives of the study were sufficiently addressed through the narrative systematic review. In addition, the heterogeneity among the included studies was too large.

## 3 Results

### 3.1 Search results

**Fig. 1:**
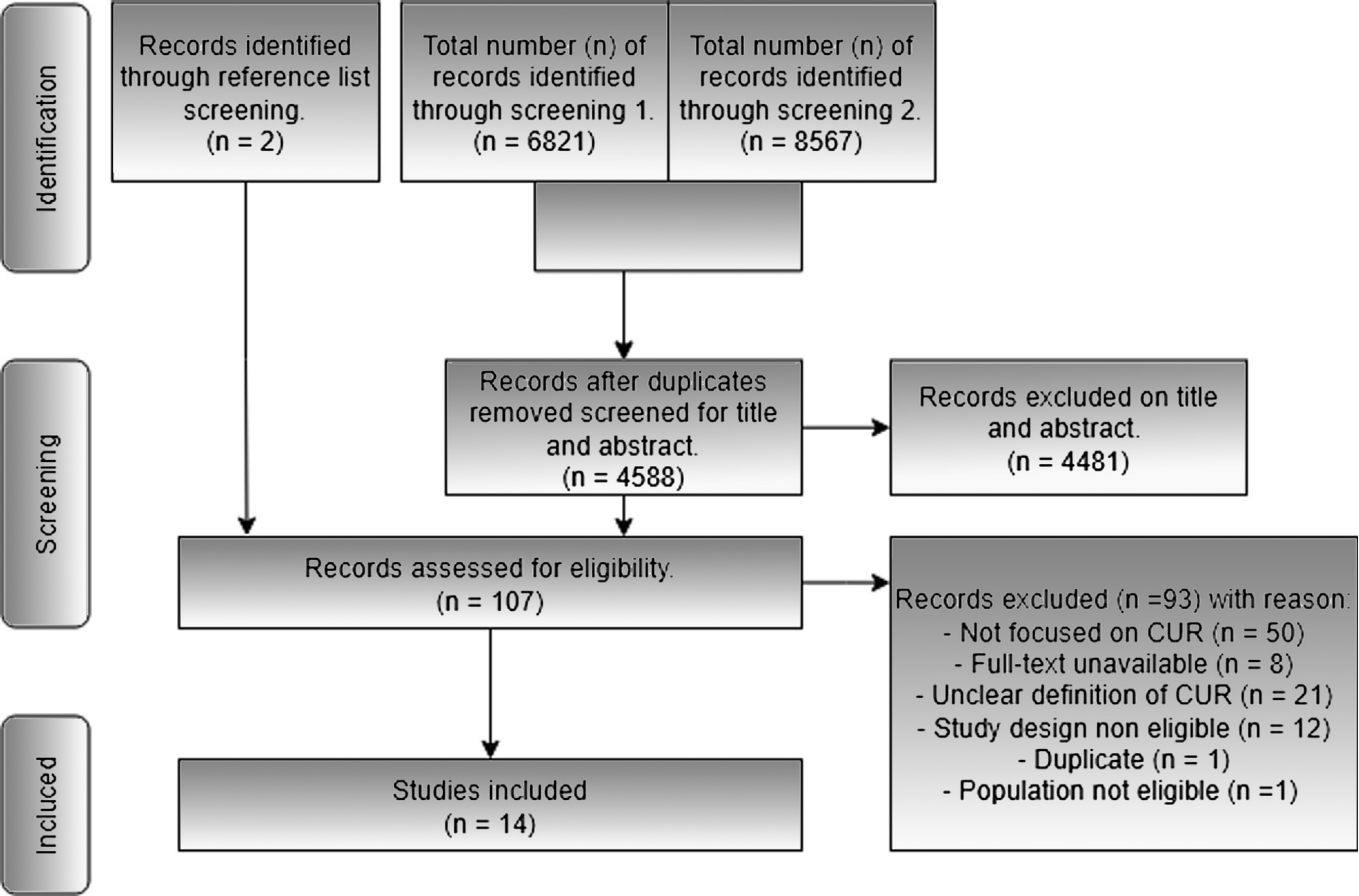
Flowchart of the screening process based on the Preferred Reporting Items for Systematic reviews and Meta-Analyses criteria. CUR = chronic urinary retention.

In total, 15 388 studies were imported for screening in the two searches. After duplicates were removed, 4588 studies were screened by abstract. One hundred seven full-text articles were screened, resulting in 14 included studies (Flowchart) (see Fig 1).

### 3.2 Study characteristics and quality assessment

Ten studies were retrospective cohort studies [8], [9], [10], [11], [12], [13], [14], [15], [16], [17], one was a cross-sectional study [18], one was a prospective cohort study [19], one was a systematic review [20], and one was a randomized controlled trial [21]. Studies were published between 1999 and 2024. The number of participants, where reported, ranged from 28 to 903. Eleven studies included male patients with CUR caused by lower urinary tract obstruction due to benign prostatic hyperplasia (BPH) [8], [9], [10], [11], [13], [14], [15], [16], [17], [20], [21], one included hospitalized older female patients with CUR [18], one included male patients with CUR and advanced prostate cancer [19], and one included male patients with a PVR >150 ml [12] (Table 1).

**Table 1:** Characteristics of the included studies

**Study type**	**Author**	**Year**	**Country**	**Type of study**	**Number of patients**	**Inclusion period**	**Age of patients with CUR (yr)**	**PVR baseline**	**Follow-up**
**Cohort study**	Alhelal et al	2024	Canada	Retrospective cohort	368	10/2017–07/2022	Median (IQR): 75 (5–96) (NCUR+NNCUR)	Median (IQR): 657 ml (300–2600)	12 mo
							NCUR: 77 (60–92), NNCUR: 75 (55-95)	NCUR: 628 ml (300–2600), NNCUR: 638 ml (370–2300)	
	MacDonald et al	2024	Canada	Retrospective cohort	168	05/2018–07/2022	Median (IQR): 77 (64–87)	Median (IQR): 1000 ml (500–1250)	12 mo
	Antoniou et al	2023	UK	Retrospective cohort	147	2012–2020	Mean (SD): 84 (3)	0–499 ml: 21	12 mo
								500–999 ml: 32	
								1000–1499 ml: 8	
								>1500 ml: 9	
								Missing: 77	
	Burton et al	2023	USA	Retrospective cohort	113	05/2021–08/2022	Median (IQR): 71 (51–90)	Mean (SD): 427 ml (147)	>6 mo
	Bos et al	2022	Netherlands	Retrospective cohort	177	01/2014–09/2020	Median (IQR): 77 (44–94)	Median (IQR): 332 ml (150–1200)	Median 68 mo
	Yuk et al	2023	South Korea	Retrospective cohort	903	01/2010–12/2016	Mean (SD): 68 (8)	Mean: 333 ml	6 mo
	Aho et al	2021	UK	Retrospective cohort	500	07/2004–03/2010	Median (IQR): 72 (67–79)	Median (IQR): 1475 ml (1200–1650)	3 mo
	Abello et al	2018	USA	Retrospective cohort	28	2002–2016	Mean (SD): 74.2 (13)	Mean (SD): 669 ml (463)	Median 56 mo
	Sood et al	2017	India	Prospective cohort	101	09/2014–04/2016	Mean (SD): 74.5 (7)	N/R	3 mo
	Jaeger et al	2015	USA	Retrospective cohort	103	2009– 2012	HoLEP median (IQR): 71 (62–77)	HoLEP median (IQR): 555 ml (390–700)	6 mo
							PVP median (IQR): 70 (62–76)	PVP median (IQR): 473 ml (327–628)	
	Reynard et al	1999	UK	Retrospective cohort	379	N/R	Successful TWOC median (IQR): 71 (43–89)	Median (IQR): 1422 ml (500–3000)	N/R
							Failed to void median (IQR): 74 (53–91)		
**Cross-sectional**	Justo et al	2017	Israel	Cross-sectional	202	N/R	Mean (SD): 85 (6)	Mean (SD): 353 (155)	1 mo
**RCT**	Ghalayini et al	2005	Jordan	RCT	41	N/R	TURP mean (SD): 67 (7)	Mean (SD): 963 (503)	6 mo
**Systematic review**	Karavitakis et al	2019	Greece	Systematic review	N/R	N/R	–	–	–

CUR = Chronic urinary retention, PVR = Postvoid residual, IQR = Inter quartile range, NCUR = Neurogenic chronic urinary retention, NNCUR = Non-neurogenic chronic urinary retention, SD = Standard deviation, HoLEP = Holium laser enucleation of the prostate, PVP = Photoselective vaporization of the prostate, N/R = Not reported, TWOC = Trial without catheter, TURP = Trans urethral resection of the prostate.

### 3.3 CUR in males with lower urinary tract obstruction due to BPH

Eleven studies focused on male patients with CUR due to BPH. Six studies defined CUR after voiding, with PVR thresholds ranging from >200 ml to >500 ml [8], [9], [10], [11], [14], [17]. Three of these studies also defined CUR in patients unable to void, all with a PVR >1000 ml in the absence of pain [8], [9], [14]. Six studies defined CUR without specifying voiding status, all using PVR thresholds of >300 ml [12], [13], [15], [16], [20], [21]. Six studies provided a median age of the participants, ranging from 70 to 77 yr [8], [9], [11], [14], [16], [17]. Four studies provided a mean age of the participants, ranging from 67 to 75 yr [10], [13], [15], [21]. Six studies provided median PVR at baseline, ranging from 332 ml to 1475 ml [8], [9], [14], [16], [17]. Four studies provided mean PVR at baseline, ranging from 333 ml to 963 ml [11], [13], [15], [21] (Table 2).

**Table 2:** Chronic urinary retention definition table

**Author**	**Condition**	**Definition CUR**	**PVR threshold**	**Voiding status defined**	**Number of times measured**	**Measurement method PVR**	**The CUR definition used**
Alhelal et al	BPH	PVR > 300 ml able to void / PVR > 1000 ml unable to void	300 ml	Yes	1	N/R	Stoffel, Aho
MacDonald et al	BPH	PVR >300 ml able to void / PVR > 1000 ml unable to void	300 ml	Yes	1	N/R	Aho
Antoniou et al	BPH and >80 yr of age	Three failed attempts at catheter removal (PVR > 300 ml) or high-pressure retention (large residual bladder volumes with radiological or biochemical evidence of reduced renal function as a result	300 ml	Yes	3	N/R	N/R
Burton et al	BPH	Spontaneously voiding and PVR > 300 ml	300 ml	Yes	1	UDS	Negro, Abrams
Bos et al	PVR > 150 ml and indication to start treatment	PVR > 150 ml measured twice in min 6 mo.	150 ml	No	2 in at least 6 mo	N/R	N/R
Yuk et al	BPH	PVR >300 ml	300 ml	No	1	N/R	Abrams
Aho et al	BPH	PVR >300 ml able to void / PVR > 1000 ml unable to void	300 ml	Yes	1	N/R	Negro
Abello et al	BPH	PVR > 300 ml 2x in at least 6 mo	300 ml	No	2 times in at least 6 mo	US or Cat	Stoffel
Sood et al	Advanced prostate cancer	PVR >200 ml	200 ml	No	1	US	N/R
Jaeger et al	BPH	Persistent PVR >300 ml or refractory urinary retention after multiple failed voiding trials, making patients catheter-dependent	200 ml	No	1	US	N/R
Reynard et al	BPH	PVR >500 ml with or without upper tract dilation on ultrasound and/or uremia occurring in a patient who was still able to void spontaneously	500 ml	Yes	1	US or Cat	Mitchell
Justo et al	Women >75 yr	PVR ≥ 200 ml without LUTS or abdominal pain, in two consecutive measurements	200 ml	No	2	Bladder scan	N/R
Ghalayini et al	BPH	PVR >300 ml measured twice	300 ml	No	2	US	Abrams
Karavitakis et al	BPH	PVR ≥ 300 ml	300 ml	No	–	–	–

CUR = Chronic urinary retention, PVR = Postvoid residual, BPH = Benign prostatic hyperplasia, N/R = Not reported, UDS = Urodynamics, US = Ultrasound, Cat = Catheterization, LUTS = Lower urinary tract symptoms.

### 3.4 CUR in hospitalized older women

One study focused on female patients, aged 75 yr and older, who were admitted for any medical indication to the Internal Medicine department with CUR [18]. CUR was defined as a PVR ≥ 200 ml without LUTS or abdominal pain after voiding in two consecutive measurements. The mean age of female patients with CUR was 85 (SD = 6.4) yr. The mean PVR of female patients with CUR at baseline was 353 (SD = 155) (Table 2).

### 3.5 CUR in male patients with advanced prostate cancer

One study focused on male patients with advanced prostate cancer with a PVR >200 ml who had not received previous treatment [19]. The voiding status was not specified. CUR was defined as a PVR >200 ml. The mean age of male patients was 75 (SD = 7) yr. The mean baseline PVR was not reported (Table 2)

### 3.6 CUR in male patients with a PVR > 150 ml

One study included male patients aged >18 yr with a PVR of >150 ml and an indication to start treatment because of bothersome symptoms and/or a high risk of complications [12]. CUR was defined as a PVR >150 ml, measured twice at least 6 mo apart. The voiding status was not specified. The median age was 77 (IQR = 44–77). The median PVR at baseline was 332 ml (IQR = 150–1200) (Table 2).

### 3.7 Measurement method

Four studies measured the PVR on two separate occasions before diagnosing CUR [12], [15], [18], [21]. Of these, two studies explicitly required a 6-mo interval between measurements [12], [18]. One study measured PVR on three separate occasions without specifying the interval between measurements [10]. The remaining nine studies did not specify the frequency of PVR measurement required to define CUR [8], [9], [11], [13], [14], [16], [17], [19].

Three studies used ultrasound (US) to measure PVR [16], [19], [21], one study used a bladder scan [18], one study used catheterization [14], two studies used US or catheterization [15], [17], and one study used urodynamics [11]. The remaining five studies did not report the measurement method [8], [9], [10], [12], [13] (Table 2).

## 4 Discussion

This systematic-narrative review provides a structured overview of how CUR is defined in the literature. A thorough search of online medical databases was conducted to ensure the inclusion of all relevant studies. The findings illustrate that there are no consensus criteria about PVR thresholds to define CUR. Although 10 out of 14 studies used a PVR threshold of >300 ml to define CUR, the evidence was limited and heterogeneous, with substantial variation in study populations, voiding status, and measurement methods.

Several articles have been published about the definition of CUR. Stoffel et al published an AUA whitepaper on the definition of non-neurogenic CUR (NNCUR) [3]. The workgroup based its decision on a comparative effectiveness review and an updated search conducted in March 2016 [22]. CUR was defined as “an elevated PVR of greater than 300 ml that has persisted for at least 6 mo and is documented on two or more separate occasions”. In the current review, only Abello et al used this definition [15]. Alhelal et al cited the AUA whitepaper but used a slightly different definition [8].

Negro et al also published a paper on the definition of CUR [23]. They performed a MEDLINE search and concluded that the definition of CUR is imprecise and arbitrary. They state that most studies seem to describe the condition as either a PVR >300 ml in men who are voiding or >1000 ml in men who are unable to void. However, this definition does not include multiple measurements and therefore does not account for the chronic aspect of the condition. Two studies based their CUR definition on this article [11], [14].

Abrams et al, as a part of the standardization subcommittee of the International Continence Society, also published an article about the definition of CUR [24]. They define it as: “a nonpainful bladder, which remains palpable or percussible after the patient has passed urine. Such patients may be incontinent.” This definition does not include a PVR threshold or multiple measurements. The authors state that a minimum PVR of 300 ml has been previously mentioned, as this is the minimal volume at which the bladder is palpable. The European Association of Urology guidelines also adopt this definition. Three studies based their CUR definition on this article [11], [13], [21].

Mitchell published a review article on the management of CUR [25]. He defines CUR as “the residual urine remaining in the bladder after micturition has reached a volume equal to or greater than the normal bladder capacity”. This definition also does not include a PVR threshold. Reynard et al based their decision on this article [17].

Eight of 14 studies used an established PVR threshold for their CUR definition, with four different definitions used. In contrast, six studies did not reference any existing PVR threshold. This underscores the heterogeneity in definitions and PVR thresholds to define CUR, highlighting its arbitrary, inconsistent, and imprecise nature.

In the most recent International Continence Society (ICS) guidelines, the CUR definition was updated in alignment with the AUA white paper proposed by Stoffel et al, reflecting international consensus between these two leading professional organizations [26]. Although the supporting evidence is acknowledged as limited, this consensus provides a valuable foundation for standardizing CUR reporting and advancing future research.

The definition proposed by Stoffel et al, established by an expert workgroup informed by a systematic review, is the most recent and comprehensive. It is the only definition that takes into account the chronic aspect of the condition by performing multiple measurements. Stoffel et al further define two categories of NNCUR: high vs low risk and symptomatic vs asymptomatic CUR. High-risk patients are those with CUR who are potentially at risk for organ system harm or failure due to retention. Symptomatic CUR is defined as: (1) having subjectively moderate to severe urinary symptoms impacting quality of life and/or, (2) having a history of requiring catheterization for the treatment of a symptomatic episode of inability to void within the last 6 mo. Based on these categorizations, a different treatment algorithm is proposed. High-risk or symptomatic CUR should be treated with catheterization and assessment of the condition, whereas low- risk and asymptomatic CUR can be monitored. However, the definition by Stoffel et al did not include patients with NCUR. In this review, the majority of studies also did not include patients with NCUR. Only Alhelal et al reported on different subgroups (NCUR, NNCUR), but they did not use different PVR thresholds for these groups. This presents a significant issue because patients with neurogenic bladder are at greater risk of high-pressure retention and other complications [27], The distinct pathophysiology and risk profile of NCUR warrants a separate, risk-stratified definition for NCUR compared with NNCUR.

Although UR is much more common in men, with an estimated male-to-female ratio of 13 to 1, primarily due to BPH, up to one-third of older women will have incomplete bladder emptying with increased PVR [28], [29]. In this review, only Justo et al included female patients [18]. An increased PVR may worsen LUTS and may be associated with a higher UTI risk [30]. Since female patients have a predisposed higher UTI risk compared with male patients, a more cautious approach may be required.

Where reported, most studies measured PVR using a bladder scan or US, both of which are reliable methods [5], [31]. In addition, a recent systematic review found that measurement with US compared to catheterization in patients with AUR, resulted in a decrease in adverse effects, such as UTIs, or lengthened hospital stays [32]. Although some studies use urodynamics, this method is too extensive for routine PVR measurement and is not available in all clinical settings or hospitals. Therefore, the preferred method for PVR measurement is US or bladder scan.

Although PVR is a pragmatic surrogate for measuring CUR, it is clinically imperfect. PVR does not take into account the time since the last void, the volume of the last void, or the maximum bladder capacity. In addition, several studies showed that PVR was variable in repeated measures and thus may not be useful as a tool for diagnosis and treatment follow-up [33], [34], [35]. A more robust measurement method that has been proposed is bladder voiding efficiency (BVE), calculated as the voided volume/ (voided volume + PVR) × 100 [36]. Yono et al demonstrated that the variability of BVE was significantly smaller compared with that of PVR [37]. Further research in larger cohorts and different patient populations is necessary to unravel the added value of BVE measurement.

This systematic-narrative review has several limitations. First, despite screening over 4000 abstracts, only 14 studies met the inclusion criteria, reflecting the scarcity of studies that explicitly define CUR using a PVR threshold. By restricting inclusion to studies that operationalize CUR with a PVR threshold, we likely excluded studies describing similar clinical entities under different terminology. However, this was a deliberate methodological choice, as PVR is widely accessible in clinical practice, making PVR-based definitions directly applicable to clinical decision-making. This highlights a broader issue: chronic UR is frequently described in the literature but rarely defined in a standardized and reproducible manner. Second, substantial heterogeneity was found between the identified studies in terms of study populations, underlying pathophysiology, voiding status, and PVR measurement methods, limiting the quality and comparability of the evidence.

The current literature does not provide empirical justification for any specific PVR threshold to define CUR. Future research should focus on standardized, patient-specific definitions of CUR that take individual risk factors into account. The measurement method should be consistent and easy accessible in the clinic. This could allow for more accurate identification of patients at risk for treatment initiation, improve consistency in reporting, and facilitate future urological research.

## 5 Conclusion

This is the first review that has evaluated the definition of CUR, focusing on PVR thresholds, in the literature. It highlights the substantial variability in definitions and PVR thresholds used to define CUR. Although a PVR threshold of ≥300 ml was most frequently used, the heterogeneity in study populations and variability in the number of measurements and measurement method resulted in limited and heterogeneous evidence. Therefore, no definitive definition of CUR could be established. A standardized, patient-specific definition of CUR that takes individual risk factors into account could improve treatment algorithm and facilitate future urological research.

  ***Author contributions***: C. H.H. Christiaans and J. de Klerk had full access to all the data in the study and takes responsibility for the integrity of the data and the accuracy of the data analysis.

  *Study concept and design*: Christiaans, de Klerk, van Velsen, Blok.

*Acquisition of data*: Christiaans, de Klerk, van Velsen.

*Analysis and interpretation of data*: Christiaans, de Klerk, van Velsen.

*Drafting of the manuscript*: Christiaans, de Klerk.

*Critical revision of the manuscript for important intellectual content*: Blok.

*Statistical analysis*: Christiaans.

*Obtaining funding*: None.

*Administrative, technical, or material support*: None.

*Supervision*: Blok.

*Other* (specify): None.

  ***Financial disclosures:*** C.H.H. Christiaans certifies that all conflicts of interest, including specific financial interests and relationships and affiliations relevant to the subject matter or materials discussed in the manuscript (eg, employment/affiliation, grants or funding, consultancies, honoraria, stock ownership or options, expert testimony, royalties, or patents filed, received, or pending), are the following: None.

  ***Funding/Support and role of the sponsor*:** None.

## Appendix A Supplementary material

Supplementary Data 1 Full search string.

## References

[1] D’Ancona C., Haylen B., Oelke M. (2019). The International Continence Society (ICS) report on the terminology for adult male lower urinary tract and pelvic floor symptoms and dysfunction. Neurourol Urodyn.

[2] Gravas S., Gacci M., Gratzke C. (2023). Summary paper on the 2023 European Association of Urology guidelines on the management of non-neurogenic male lower urinary tract symptoms. Eur Urol.

[3] Stoffel J.T., Peterson A.C., Sandhu J.S., Suskind A.M., Wei J.T., Lightner D.J. (2017). AUA white paper on nonneurogenic chronic urinary retention: consensus definition, treatment algorithm, and outcome end points. J Urol.

[4] Mindardi D. Urinary retention. 2018. ICS. https://www.ics.org/committees/standardisation/terminologydiscussions/urinaryretention.

[5] Asimakopoulos A.D., De Nunzio C., Kocjancic E., Tubaro A., Rosier P.F., Finazzi-Agrò E. (2016). Measurement of post-void residual urine. Neurourol Urodyn.

[6] Turnbull D., Chugh R., Luck J. (2023). Systematic-narrative hybrid literature review: a strategy for integrating a concise methodology into a manuscript. Soc Sci Humanit Open.

[7] Page M.J., McKenzie J.E., Bossuyt P.M. (2021). The PRISMA 2020 statement: an updated guideline for reporting systematic reviews. BMJ.

[8] Alhelal S., Nikoufar P., Hodhod A. (2024). Efficacy and durability of holmium laser enucleation of the prostate (HoLEP) in the management of acute and chronic urinary retention: a retrospective study. Can Urol Assoc J.

[9] MacDonald A., Fathy M., Nikoufar P. (2023). Efficacy of GreenLight laser prostatectomy in urinary retention. Can Urol Assoc J.

[10] Antoniou V., Edris F., Akpobire W., Voss J., Somani B. (2023). Surgical outcomes for elderly patients undergoing transurethral resection of the prostate for chronic urinary retention and proposal of a management algorithm. J Endourol.

[11] Burton C.S., Dobberfuhl A.D., Comiter C.V. (2023). Outcomes of aquablation in men with acute and chronic urinary retention. Urology.

[12] Bos B.J., van Merode N.A.M., Steffens M.G., Witte L.P.W. (2022). The patient pathway for men with chronic urinary retention: treatments, complications, and consequences. Urology.

[13] Yuk H.D., Oh S.J. (2023). Effect of urinary retention on the surgical outcome of holmium laser enucleation of the benign prostatic hyperplasia. Investig Clin Urol.

[14] Aho T., Finch W., Jefferson P., Suraparaju L., Georgiades F. (2021). HoLEP for acute and non-neurogenic chronic urinary retention: how effective is it?. World J Urol.

[15] Abello A., DeWolf W.C., Das A.K. (2019). Expectant long‐term follow‐up of patients with chronic urinary retention. Neurourology and Urodynamics.

[16] Jaeger C.D., Mitchell C.R., Mynderse L.A., Krambeck A.E. (2015). Holmium laser enucleation (HoLEP) and photoselective vaporisation of the prostate (PVP) for patients with benign prostatic hyperplasia (BPH) and chronic urinary retention. BJU Int.

[17] Reynard J.M., Shearer R.J. (1999). Failure to void after transurethral resection of the prostate and mode of presentation. Urology.

[18] Justo D., Schwartz N., Dvorkin E., Gringauz I., Groutz A. (2017). Asymptomatic urinary retention in elderly women upon admission to the Internal Medicine department: a prospective study. Neurourol Urodyn.

[19] Sood R., Singh R.K., Goel H., Manasa T., Khattar N., Tripathi M.C. (2017). Can androgen-deprivation therapy obviate the need of channel transurethral resection of the prostate in advanced prostate cancer with urinary retention? A prospective study. Arab J Urol.

[20] Karavitakis M., Kyriazis I., Omar M.I. (2019). Management of urinary retention in patients with benign prostatic obstruction: a systematic review and meta-analysis. Eur Urol.

[21] Ghalayini I.F., Al-Ghazo M.A., Pickard R.S. (2005). A prospective randomized trial comparing transurethral prostatic resection and clean intermittent self-catheterization in men with chronic urinary retention. BJU Int.

[22] Brasure M., Fink H.A., Risk M. (2014). Comparative effectiveness review.

[23] Negro C.L.A., Muir G.H. (2012). Chronic urinary retention in men: how we define it, and how does it affect treatment outcome. BJU Int.

[24] Abrams P., Cardozo L., Fall M. (2003). The standardisation of terminology in lower urinary tract function: report from the standardisation sub-committee of the International Continence Society. Urology.

[25] Mitchell J.P. (1984). Management of chronic urinary retention. Br Med J (Clin Res Ed).

[26] D'Ancona C., Haylen B., Oelke M. (2019). The International Continence Society (ICS) report on the terminology for adult male lower urinary tract and pelvic floor symptoms and dysfunction. Neurourol Urodyn.

[27] Sartori A.M., Kessler T.M., Castro-Díaz D.M. (2024). Summary of the 2024 update of the European Association of Urology guidelines on neuro-urology. Eur Urol.

[28] Leslie S.W., Rawla P., Dougherty J.M. (2025).

[29] Grosshans C., Passadori Y., Peter B. (1993). Urinary retention in the elderly: a study of 100 hospitalized patients. J Am Geriatr Soc.

[30] Arlandis S., Bø K., Cobussen-Boekhorst H. (2022). European Association of Urology guidelines on the management of female non-neurogenic lower urinary tract symptoms. Part 2: Underactive bladder, bladder outlet obstruction, and nocturia. Eur Urol.

[31] Schallom M., Prentice D., Sona C. (2020). Accuracy of measuring bladder volumes with ultrasound and bladder scanning. Am J Crit Care.

[32] Fernández-Prada I., Ballesteros-Peña S. (2025). Aplicación de la ecografía vesical para reducir sondajes en pacientes con sospecha de retención aguda de orina: una revisión sistemática. Enfirm Clin.

[33] Saaby M.L., Lose G. (2012). Repeatability of post-void residual urine ≥ 100 ml in urogynaecologic patients. Int Urogynecol J.

[34] Bruskewitz R.C., Iversen P., Madsen P.O. (1982). Value of postvoid residual urine determination in evaluation of prostatism. Urology.

[35] Griffiths D.J., Harrison G., Moore K., McCracken P. (1996). Variability of post-void residual urine volume in the elderly. Urol Res.

[36] Abrams P. (1999). Bladder outlet obstruction index, bladder contractility index and bladder voiding efficiency: three simple indices to define bladder voiding function. BJU Int.

[37] Yono M., Ito K., Oyama M. (2021). Variability of post-void residual urine volume and bladder voiding efficiency in patients with underactive bladder. Low Urin Tract Symptoms.

