# Supplementary material for: Postvoid Residual Thresholds Used to Define Chronic Urinary Retention: A Systematic Review
Source: Eur Urol Open Sci. 2026 Jul 3;90:84–91. doi: 10.1016/j.euros.2026.06.010 (PMC13355490; doi:10.1016/j.euros.2026.06.010)
Supplement: Supplementary Data 1 — Full search string. [file mmc1.docx]

**Supplementary material**

**Complete search strategy**

| **Database searched** | **Platform** | **Years of coverage** | **Records** | **Records after duplicates removed** |
| --- | --- | --- | --- | --- |
| Medline ALL | Ovid | 1946 - Present | 2334 | 2320 |
| Embase | Embase.com | 1971 - Present | 3194 | 1262 |
| Web of Science Core Collection* | Web of Knowledge | 1975 - Present | 2334 | 492 |
| Cochrane Central Register of Controlled Trials | Wiley | 1992 - Present | 987 | 656 |
| Additional Search Engines: Google Scholar** (200 top-ranked) | | | 200 | 115 |
| **Total** | | | **9049** | **4845** |

*Science Citation Index Expanded (1975-present) ; Social Sciences Citation Index (1975-present) ; Arts & Humanities Citation Index (1975-present) ; Conference Proceedings Citation Index- Science (1990-present) ; Conference Proceedings Citation Index- Social Science & Humanities (1990-present) ; Emerging Sources Citation Index (2005-present)

**Google Scholar was searched via "Publish or Perish" to download the results in EndNote.

No other database limits were used than those specified in the search strategies

**Embase**

('urine retention'/de OR 'postvoid residual urine volume'/de OR 'urodynamics'/de OR 'dysfunctional voiding'/de OR (((urin*) NEAR/3 (retent*)) OR ((post-void* OR postvoid*) NEAR/3 (residue* OR residual* OR bladder-vol*)) OR urodynamic* OR ((dysfunctional) NEAR/3 (voiding)) OR NNCUR):ab,ti,kw) **AND** ('definition'/de OR 'abnormal value'/de OR 'consensus development'/de OR 'practice guideline'/mj/de OR 'good clinical practice'/mj/de OR 'standardization'/mj/de OR 'cut off value'/de OR 'threshold'/de OR 'reference value'/mj/de OR 'benchmarking'/mj/de OR 'consensus'/mj/de OR 'palpation'/de OR 'percussion'/de OR (high-retent* OR ((abnormal* OR significant* OR large* OR elevat* OR increased OR definition* OR define* OR cut-off* OR cutoff* OR threshold* OR referenc* OR benchmark* OR consensus OR white-paper* OR best-practice* OR ml OR palpat* OR palpabl* OR percussion* OR percussab*) NEAR/3 (retention* OR post-void* OR postvoid* OR PVR OR PVRV OR voiding OR NNCUR OR UR OR CUR)) OR good-uro*-practice*):ab,ti,kw OR (guideline* OR good-clinical-practice* OR standardi* OR ((reference) NEAR/3 (value*)) OR benchmark* OR consensus):ti) NOT ('child'/exp NOT ('adult'/exp OR 'adolescent'/de)) NOT (([Conference Abstract]/lim OR [Conference Review]/lim) AND [1800-2019]/py) NOT ('case report'/de OR (case-report):ti) NOT ([animals]/lim NOT [humans]/lim) AND [ENGLISH]/lim

**Medline**

(Urinary Retention/ OR Urodynamics/ OR (((urin*) ADJ3 (retent*)) OR ((post-void* OR postvoid*) ADJ3 (residue* OR residual* OR bladder-vol*)) OR urodynamic* OR ((dysfunctional) ADJ3 (voiding)) OR NNCUR).ab,ti,kf.) **AND** (Urinary Retention/cl OR Urinary Retention/sn OR Consensus/ OR Development Conferences as Topic/ OR Consensus Development Conferences as Topic.pt OR Practice Guideline.pt OR Practice Guidelines as Topic/ OR Reference Standards/ OR *Reference Values/ OR *Benchmarking/ OR Palpation/ OR Percussion/ OR (high-retent* OR ((abnormal* OR significant* OR large OR elevated OR increased OR definition* OR define* OR cut-off* OR cutoff* OR threshold* OR reference OR benchmark* OR consensus OR white-paper* OR best-practice* OR ml OR palpat* OR palpabl* OR percussion* OR percussab*) ADJ3 (retention* OR post-void* OR postvoid* OR PVR OR PVRV OR voiding OR NNCUR OR UR OR CUR)) OR good-uro*-practice*).ab,ti,kf. OR (guideline* OR good-clinical-practice* OR standardi* OR ((reference) ADJ3 (value*)) OR benchmark* OR consensus).ti.) NOT ((exp Child/ OR exp Infant/) NOT (exp Adult/ OR exp Adolescent/)) NOT ((news OR congres* OR abstract* OR book* OR chapter* OR dissertation abstract*).pt. AND 1800:2019.(sa_year).) NOT (Case Reports/ OR (case-report).ti.) NOT (exp animals/ NOT humans/) AND english.la.

**Cochrane**

((((urin*) NEAR/3 (retent*)) OR ((post NEXT void* OR postvoid*) NEAR/3 (residue* OR residual OR bladder NEXT vol*)) OR urodynamic* OR ((dysfunctional) NEAR/3 (voiding)) OR NNCUR):ab,ti) **AND** ((high NEXT retent* OR ((abnormal* OR significant* OR large OR elevated OR increased OR definition* OR define* OR cut NEXT off* OR cutoff* OR threshold* OR reference OR benchmark* OR consensus OR white NEXT paper* OR best NEXT practice* OR ml OR palpat* OR palpabl* OR percussion* OR percussab*) NEAR/3 (retention* OR post NEXT void* OR postvoid* OR PVR OR PVRV OR voiding OR NNCUR OR UR OR CUR)) OR good NEXT uro* NEXT practice*):ab,ti OR (guideline* OR good NEXT clinical NEXT practice* OR standardi* OR ((reference) NEAR/3 (value*)) OR benchmark* OR consensus):ti) NOT (((child* OR schoolchild* OR minors OR infan* OR toddler* OR pediatr* OR paediatr* OR baby OR babies OR girl* OR boy* OR newborn* OR neonate* OR premature* OR pre NEXT matur* OR kid OR kids OR underag* OR kindergar* OR prepubesc* OR school* OR preschool* OR suckling OR PICU OR NICU OR PICUs OR NICUs):ab,ti) NOT ((adult* OR elderl* OR man OR men OR woman OR women OR frail* OR octagener* OR geriat* OR ((old*) NEAR/3 (patient* OR peopl*)) OR adolescen* OR preadolescen*):ab,ti)) NOT "conference abstract":pt

**Web of Science**

(TS=(((((urin*) NEAR/2 (retent*)) OR ((post-void* OR postvoid*) NEAR/2 (residue* OR residual OR bladder-vol*)) OR urodynamic* OR ((dysfunctional) NEAR/2 (voiding)) OR NNCUR))) **AND** (TS=((high-retent* OR ((abnormal* OR significant* OR large OR elevated OR increased OR definition* OR define* OR cut-off* OR cutoff* OR threshold* OR reference OR benchmark* OR consensus OR white-paper* OR best-practice* OR ml OR palpat* OR palpabl* OR percussion* OR percussab*) NEAR/2 (retention* OR post-void* OR postvoid* OR PVR OR PVRV OR voiding OR NNCUR OR UR OR CUR)) OR good-uro*-practice*)) OR TI=(guideline* OR good-clinical-practice* OR standardization* OR ((reference) NEAR/2 (value*)) OR benchmark* OR consensus))) NOT TS=(((child* OR schoolchild* OR minors OR infan* OR toddler* OR pediatr* OR paediatr* OR baby OR babies OR girl* OR boy* OR newborn* OR neonate* OR premature* OR pre-matur* OR kid OR kids OR underag* OR kindergar* OR prepubesc* OR school* OR preschool* OR suckling OR PICU OR NICU OR PICUs OR NICUs)) NOT ((adult* OR elderl* OR man OR men OR woman OR women OR frail* OR octagener* OR geriat* OR ((old*) NEAR/2 (patient* OR peopl*)) OR adolescen* OR preadolescen*))) NOT TS=((animal* OR rat OR rats OR mouse OR mice OR murine OR dog OR dogs OR canine OR cat OR cats OR feline OR rabbit OR cow OR cows OR bovine OR rodent* OR sheep OR ovine OR pig OR swine OR porcine OR veterinar* OR chick* OR zebrafish* OR baboon* OR nonhuman* OR primate* OR cattle* OR goose OR geese OR duck OR macaque* OR avian* OR bird* OR fish*) NOT (human* OR patient* OR women OR woman OR men OR man)) NOT (DT=(Meeting Abstract OR Meeting Summary) AND py=(1800-2019)) NOT TI=(case-report) AND LA=(English)

**Google Scholar**

"urine|urinary retention"|"postvoid residual urine" define|definition|"abnormal|significant|large|elevated|increased|cutoff|threshold|reference retention|volume|PVR|PVRV|NNCUR|UR|CUR" -child -children -animal -rat -mouse -mice

'urine|urinary retention'|'postvoid residual urine' define|definition|'abnormal|significant|large|elevated|increased|cutoff|threshold|reference retention|volume|PVR|PVRV|NNCUR|UR|CUR' -child -children -animal -rat -mouse -mice
